# Effectiveness of myopia control interventions: A systematic review of 12 randomized control trials published between 2019 and 2021

**DOI:** 10.3389/fpubh.2023.1125000

**Published:** 2023-03-23

**Authors:** Carla Lanca, Chi Pui Pang, Andrzej Grzybowski

**Affiliations:** ^1^Escola Superior de Tecnologia da Saúde de Lisboa (ESTeSL), Instituto Politécnico de Lisboa, Lisboa, Portugal; ^2^Comprehensive Health Research Center (CHRC), Escola Nacional de Saúde Pública, Universidade Nova de Lisboa, Lisboa, Portugal; ^3^Department of Ophthalmology and Visual Sciences, The Chinese University of Hong Kong, Hong Kong, China; ^4^Hong Kong Hub of Paediatric Excellence, The Chinese University of Hong Kong, Hong Kong, China; ^5^Joint Shantou International Eye Center, Shantou University/The Chinese University of Hong Kong, Shantou, China; ^6^Department of Ophthalmology, University of Warmia and Mazury, Olsztyn, Poland; ^7^Institute for Research in Ophthalmology, Foundation for Ophthalmology Development, Poznan, Poland

**Keywords:** myopia, progression, axial length, elongation, treatment, efficacy, systematic review

## Abstract

**Purpose:**

This study aims to investigate the effectiveness of interventions to control myopia progression. In this systematic review, the primary outcomes were mean differences (MD) between treatment and control groups in myopia progression (D) and axial length (AL) elongation (mm).

**Results:**

The following interventions were found to be effective (*p* < 0.001): highly aspherical lenslets (HAL, 0.80 D, 95% CI, 0.77–0.83; −0.35 mm, 95% CI −0.36 to −0.34), MiSight contact lenses (0.66 D, 95% CI, 0.63–0.69; −0.28 mm, 95% CI −0.29 to −0.27), low dose atropine 0.05% (0.54 D, 95% CI, 0.38–0.70; −0.21 mm, 95% CI-0.28 to −0.14), Biofinity +2.50 D (0.45 D, 95% CI, 0.29, 0.61; −0.24 mm, 95% CI −0.33 to −0.15), defocus incorporated multiple segments [DIMS] (0.44 D, 95% CI, 0.42–0.46; −0.34 mm, 95% CI −0.35 to −0.33) and ortho-k lenses (−0.24 mm, 95% CI −0.33 to −01.5).

**Conclusion:**

Low-dose atropine 0.01% was not effective in reducing AL progression in two studies. Treatment efficacy with low-dose atropine of 0.05% showed good efficacy. Spectacles (HAL and DIMS) and contact lenses (MiSight and Biofinity) may confer a comparable treatment benefit compared to atropine, to slow myopia progression.

## 1. Introduction

Myopia prevalence has increased worldwide and although myopia is more prevalent in East Asia, epidemiological studies show an increasing rate in European populations (1). The variations in myopia prevalence have been attributed to both genetic and environmental factors although the interactive causative effects are still to be established. Increasing intensity and duration of education are risk factors linked to higher myopia prevalence (2, 3). Increased risk of myopia has been found in children who perform more near work, spend less time outdoors and have myopic parents (4). Controlling myopia progression to avoid future high myopia and visual impairment is becoming more common in routine ophthalmology practice in some regions of the world where the prevalence is high, such as East Asian countries. The risk of developing myopic maculopathy (58%), retinal detachment (30%), posterior subcapsular cataract (21%) and open-angle glaucoma (20%) increases with each additional 1 D of myopia (5). Children with myopia are also at higher risk of developing depression compared to normally sighted children (6).

In recent years, various manuscripts (both original studies and narrative or systemic reviews) on myopia epidemiology, prevention, risk factors and myopia control, have been published. According to data from PubMed there were over 1,000 manuscripts published per year in 2019 (*n* = 1,401), 2020 (*n* = 1,686) and 2021 (*n* = 1994; www.pubmed.ncbi.nlm.nih.gov). Thus, following the knowledge developments in this field is becoming more difficult. Several treatment options for myopia control have emerged in recent years. A few meta-analyses on myopia treatment efficacy were published in 2022 (5–7). Those publications have analyzed the efficacy of individual therapies on myopia control, such as atropine (7), multifocal lens (8) or atropine and orthokeratology (9). However, those studies did not compare the overall treatment effects. Additionally, 2-year data on highly aspherical lenslets (HAL) have been published (8–10). The present study updates the published evidence by comparing the efficacy of known treatments with HAL. This information may be useful to facilitate decision-making in clinical practice, especially to assist eye care providers in the choice of treatment for myopia control.

This review aims to investigate the effectiveness of interventions to control myopia progression. We present an overview of the manuscripts published between 2019 and 2021, as well as recent and relevant contributions to this important area of ophthalmology. Additionally, this study compares the efficacy in myopia control among different myopia control therapies.

## 2. Materials and methods

In this review, randomized control trials (RCT) were included if they compared interventions for slowing myopia progression in children with a treatment duration of at least 1 year. The primary outcomes of this study were the mean differences between treatment and control groups in myopia progression (D) and axial length elongation (mm) for the longer follow-up time reported in the RCT. The inclusion criteria were as follows: (1) RCT; (2) studies on treatment for myopia control published between 2019 and 2021; (3) children with myopia aged <18 years; (4) follow-up period of 1 year or more; (5) studies written in English language. Studies were excluded if (1) they had a retrospective component, were review papers or protocols, (2) they lacked the required outcome measures of this study, (3) refraction was measured without cycloplegia or not obtained using automated refraction, or (4) children were older than 16 years at baseline.

A previous Cochrane systematic review reviewed studies published up to 2018 (11). Thus, in this review we searched Pubmed, Embase and Cochrane Library publications from January 2019 to August 2021. The following search terms were selected: “Myopia AND Disease Progression NOT Keratomileusis, Laser *in Situ* NOT surgery AND humans AND Clinical Trial OR Randomized Controlled Trial OR Controlled Clinical Trial OR English Abstract OR Journal Article AND infant OR child OR adolescent.” We reviewed the references of all retrieved articles to identify articles not captured by the initial electronic search. Data was extracted and documented by one of the authors (CL) and verified by the other (AG). The Preferred Reporting Items for Systematic Reviews and Meta-Analyses (PRISMA) 2020 checklist was used. We extracted the following information from each trial: type of intervention, follow-up duration, sample size and age, mean change in refraction and axial length.

The methodological quality of RCTs was evaluated using the Cochrane Collaboration’s Risk of Bias Assessment tool (RoB v.2.0) (12). The methodology examined the following aspects of each trial: bias arising from randomization process, bias due to deviations from intended intervention, bias due to missing outcome data, bias in measurement of the outcome and bias in selection of the reported result. We graded each of the item domains as “low” and “high” risk of bias or “some concerns.”

Missing standard deviations were derived from other statistics, such as *p*-values or confidence intervals (CI), if needed (13).

A random effects analysis was performed to obtain conservative pooled estimates that took in consideration heterogeneity and sampling error. We also assessed heterogeneity with the *I*^2^ statistics. The statistical heterogeneity was considered significant when the *I*^2^ statistic was greater than or equal to 50%. Data analysis was started with a fixed-effect model and then switched to a random-effects model upon realizing the significant test of heterogeneity. The results of the different studies and the overall effect (under the random effects model) with 95% CI were illustrated with forest plots graphs. For the outcome myopia progression, a positive mean difference [MD] indicates that the intervention was better compared with the control group (less myopia progression). For the outcome axial length, a negative MD indicates that the intervention was better compared with the control group (less axial elongation). As there was variation in sample sizes across the studies and more than 10 studies were included, we assessed publication bias using the funnel plot. A *p*-value of <0.05 was accepted as statistically significant. RevMan v. 5.4 software was used for the statistical analysis.

## 3. Results

**Figure 1:**
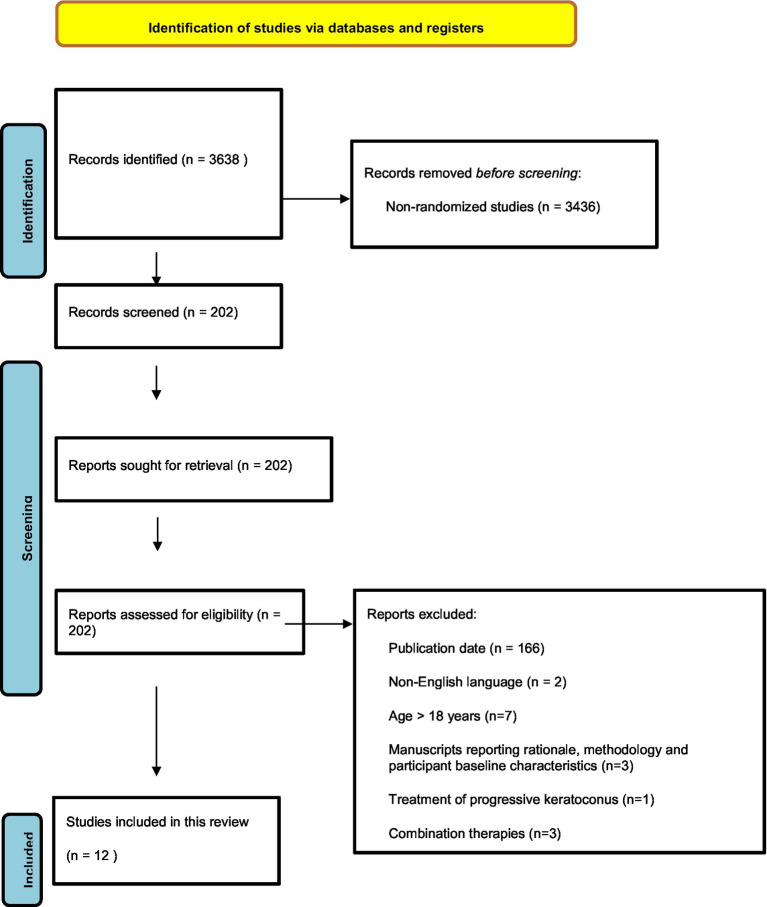
PRISMA flow diagram.

The electronic search identified a total of 3638 studies. Figure 1 presents a PRISMA flow diagram showing the process of obtaining eligible studies. A total of 3436 non-RCTs were excluded, and 202 studies were screened. After screening, 12 studies met the inclusion criteria and were included in this review (Figure 1). Among the 12 RCTs four main types of interventions to control myopia progression were found, including topical low-dose atropine eye drops (5 studies), multifocal spectacles (2 studies), multifocal contact lenses with aspheric or discrete dual-focus designs (4 studies), and overnight orthokeratology (ortho-k lenses, 1 study). The characteristics of the 12 included studies are presented in Table 1.

**Table 1:** Characteristics of RCTs included in the review (*n* = 12).

Study	Location	Study group	Control group	Follow-up (months)	Age (years)	Myopia range (D)	Side effects
Atropine							
LAMP (Yam et al.) (14–16)	China	0.05, 0.025, and 0.01%	Placebo	12^a^	4–12	<−1.00	Severe adverse events (*n* = 20), were not related to atropine therapy
I-ATOM (Saxena et al.) (17)	India	0.01%	Placebo	12	6–14	−0.50 to −6.00	No side effects were reported
ATOM-J (Hieda et al.) (18)	Japan	0.01%	Placebo	24	6–12	−1.00 to −6.00	Mild allergic conjunctivitis side effects (*n* = 2)
Fu et al. (19)	China	0.02 and 0.01%	Single-vision spectacles	12	6–14	−1.25 to −6.00	Allergy (0.01%: *n* = 1) and photophobia in bright sunlight (0.02: *n* = 32); (0.01%: *n* = 33)
Wei et al. (20)	China	0.01%	Placebo	12	6–12	−1.00 to −6.00	No serious adverse events were reported
Soft contact lenses for myopia control							
MiSight contact lenses (Chamberlain et al.) (21, 22)	Portugal, United Kingdom, Singapore, and Canada.	Dual-focus optical design	Single vision contact lenses	36^c^	8 to <13	−0.75 to −4.00	No serious adverse events were reported
Bifocal Lenses Biofinity +2.50 D (Walline et al.) (23)	United States of America	Multifocal contact lenses with high add power (+2.50 D)	Single vision contact lenses	36	7–11	−0.75 to −5.00	No serious adverse events were reported
Extended depth of focus contact lenses (Sankaridurg et al.) (24)	China	Lenses I and II: power in the periphery increased up to +2.50D and +1.50D; Lenses III and IV: extended depth of focus up to +1.75D and +1.25D	Single vision contact lenses	24	8–13	−0.75 to −3.50	A large number of children discontinued the treatment (25.4%)
Esencia lens (Garcia-del valle et al.) (25)	Spain	Progressive multifocal and reverse geometry	Single vision contact lenses	12	7–15	−0.50 to −8.75	No serious adverse events were reported
Spectacle lenses for myopia control							
DIMS spectacle lenses (Lam et al.) (26, 27)	Hong Kong	Hexagonal central zone of distance refractive correction surrounded by an annular zone with dense microlens segments of 3.50 D addition	Single vision spectacle lenses	24^b^	8–13	−1.00 to −5.00	–
Highly aspherical lenslets (Bao et al.) (10, 28, 29)	China	Volume of myopic defocus in front of the retina with 11 concentric rings of contiguous lenslets	Single vision spectacle lenses	24	8–13	−0.75 to −4.75	No serious adverse events were reported
Orthokeratology lenses							
Jakobsen and Møller (30)	Denmark	Dreamlite^®^: four-zone reverse geometry lens with a 6 mm optic zone diameter and 0.75 D compression factor	Single vision spectacle lenses	18	6–12	−0.5 to −4.75	No serious adverse events were reported (30% dropped out)

LAMP, Low-Concentration Atropine for Myopia Progression; I-ATOM, Atropine for treatment of childhood myopia in India; ATOM-J atropine in the treatment of myopia in Japan; DIMS, defocus incorporated multiple segments.

a The 24-follow-up data was not included in the meta-analysis as children in the placebo group switched to the atropine 0.05%. At 36 months study children in continued treatment group were compared with a washout subgroup.

b The 36 months follow-up data was not included in the meta-analysis as children from the control group switched to DIMS lenses in the third year.

c The 6-year follow-up data was not included in the meta-analysis.

Eleven studies reported both refraction and axial length outcomes, and 1 study only reported axial length.

Table 2 shows the quality assessment results. Overall, the RCTs included in this analysis seem to have a low to moderate risk of bias, with most of the RCTs reporting adequate random sequence generation, allocation concealment, and blinding of outcome assessment. Two studies were classified as having “some concerns” arising from the randomization process and 5 were classified as having “some concerns” (*n* = 2) or “high risk of bias” (*n* = 3) due to loss of follow-up or missing data. However, in some studies the treatment may not be completely masked due to the type of lenses or its effects, such as pupil dilation.

**Table 2:** Quality of studies included in the review (*n* = 12).

Study	Bias arising from randomization process	Bias due to deviations from intended intervention	Bias due to missing outcome data	Bias in measurement of the outcome	Bias in selection of the reported result
LAMP (Yam et al.) (14–16)	Low risk	Low risk	Low risk	Low risk	Low risk
I-ATOM (Saxena et al.) (17)	Low risk	Low risk	Low risk	Low risk	Low risk
ATOM-J (Hieda et al.) (18)	Low risk	Low risk	Low risk	Low risk	Low risk
Fu et al. (19)	Some concerns	Low risk	High risk	Low risk	Low risk
Wei et al. (20)	Some concerns	Low risk	Low risk	Low risk	Low risk
MiSight contact lenses (Chamberlain et al.) (21, 22)	Low risk	Low risk	Some concerns	Low risk	Low risk
Bifocal Lenses Biofinity +2.50 D (Walline et al.) (23)	Low risk	Low risk	Low risk	Low risk	Low risk
Extended depth of focus contact lenses (Sankaridurg et al.) (24)	Low risk	Low risk	Some concerns	Low risk	Low risk
Esencia lens (Garcia-del valle et al.) (25)	Low risk	Low risk	High risk	Low risk	Low risk
DIMS spectacle lenses (Lam et al.) (26, 27)	Low risk	Low risk	Low risk	Low risk	Low risk
Highly aspherical lenslets (Bao et al.) (10, 28, 29)	Low risk	Low risk	Low risk	Low risk	Low risk
Jakobsen and Møller (30)	Low risk	Low risk	High risk	Low risk	Low risk

Green: Low risk; Orange: Some concerns; Red: High risk.

There are some issues that should be noted, mainly related with the need to use data from intervention groups and the comparison with placebo groups: The Low-Concentration Atropine for Myopia Progression (LAMP) study was a RCT over 26 months, and we only used data from the first follow-up with 1-year treatment effects (11–13). The MiSight contact lenses study was a RCT over 6 years, and we only used data from the 36 months (14, 15). The defocus incorporated multiple segments [DIMS] spectacle lenses study was a RCT over 36 months, and we only selected data from the 2-year treatment effects (16, 21).

**Figure 2:**
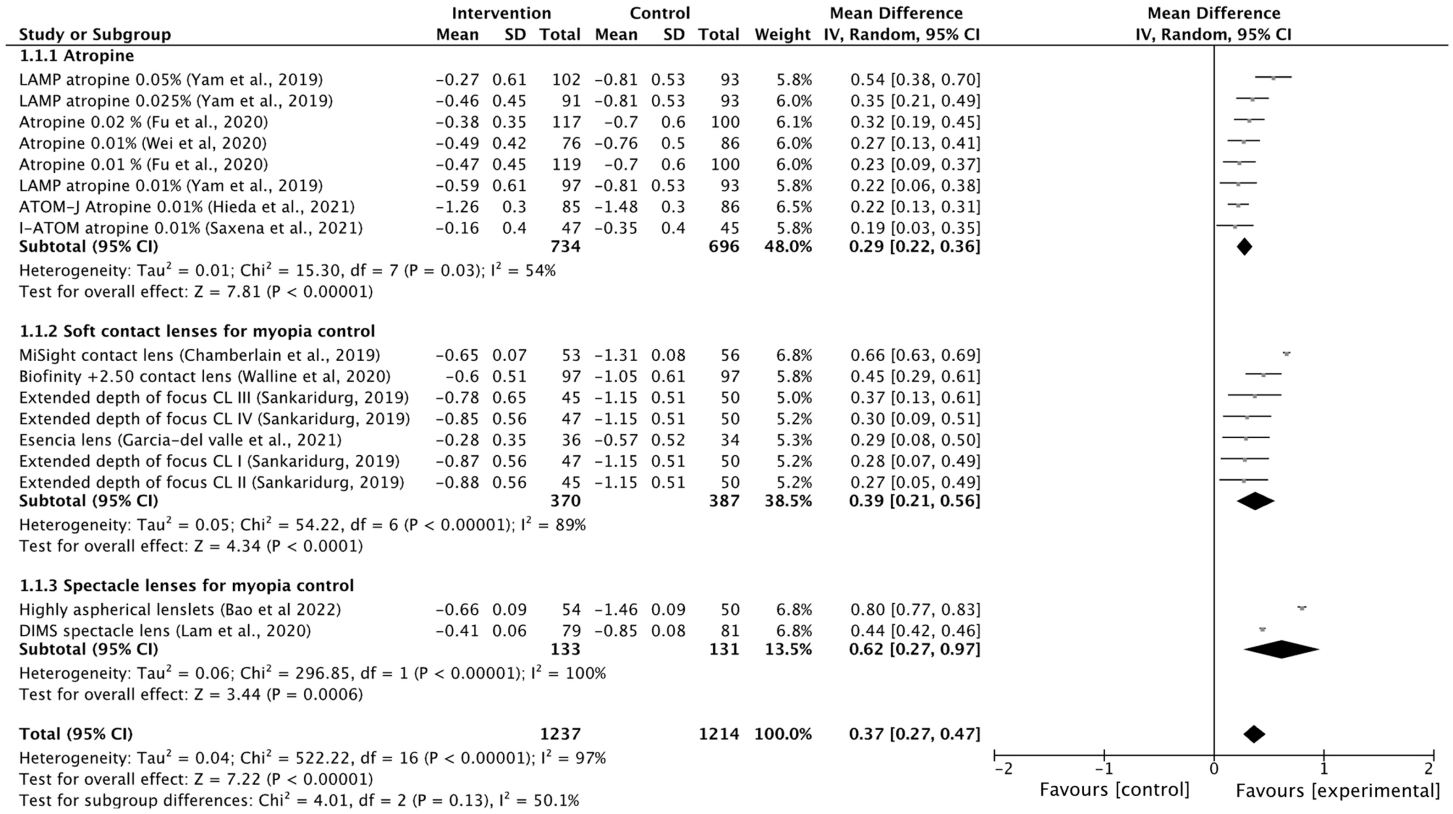
Forest plot of myopia progression (D) showing mean differences between treatment and control groups. The point estimate for the mean difference for each study is shown in gray color. The weight assigned to each study is represented by the size of each gray point estimate. The horizontal line through each gray point estimate shows the 95% confidence interval for the mean difference for each treatment. CL, contact lenses; CI, confidence interval; SD, standard deviation.

**Figure 3:**
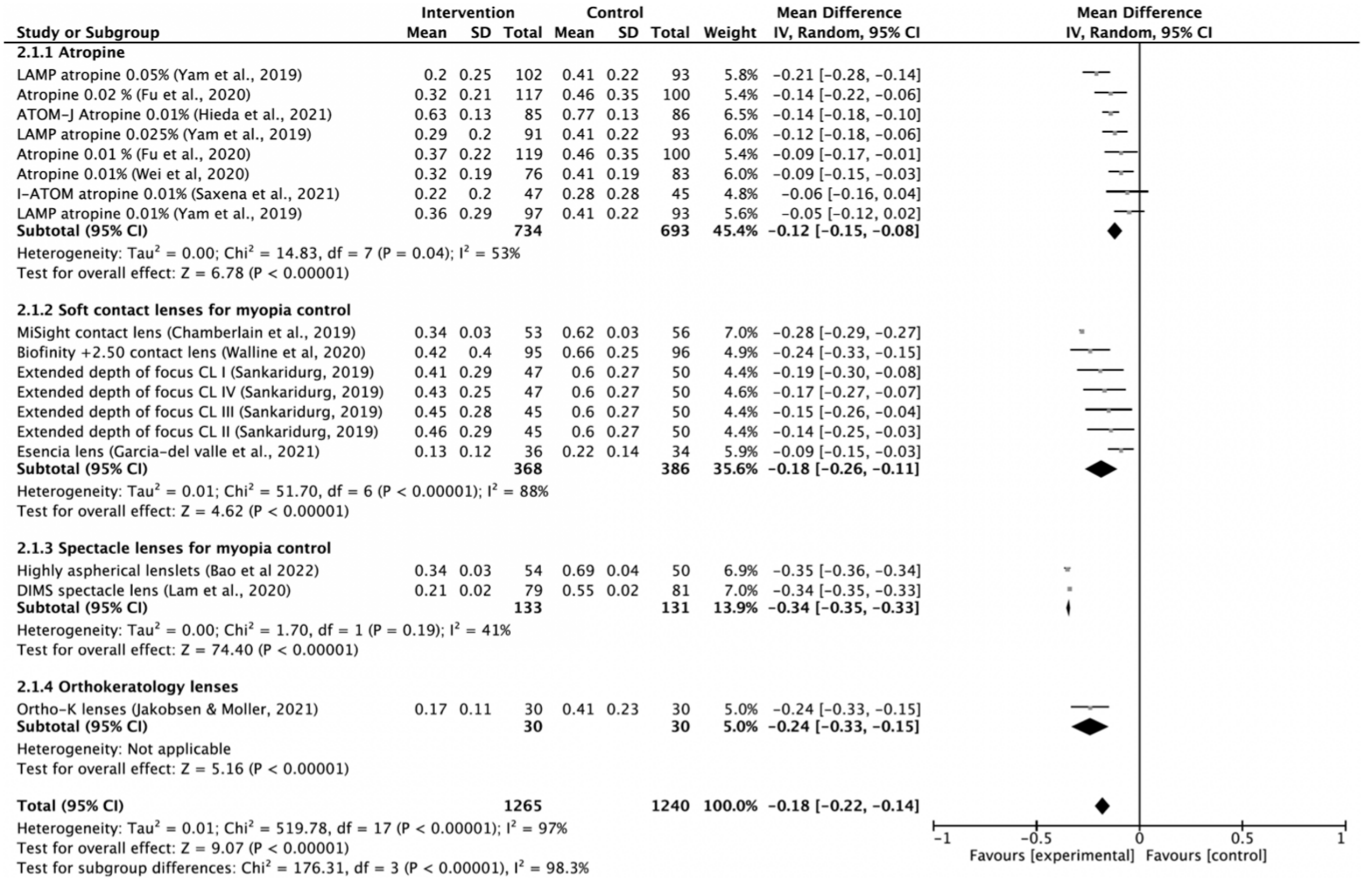
Forest plot of axial length elongation (mm) showing mean differences between treatment and control groups. The point estimate for the mean difference for each study is shown in gray color. The weight assigned to each study is represented by the size of each gray point estimate. The horizontal line through each gray point estimate shows the 95% confidence interval for the mean difference for each treatment. CL, contact lenses; CI, confidence interval; SD, standard deviation; K, keratology.

Most of the intervention methods slowed myopia progression compared to single vision spectacle lenses, single vision contact lenses or placebo. However, there were differences in treatment efficacy. The myopia progression MD for atropine was 0.29 D (95% CI 0.22, 0.36; *p* = 0.03), for soft contact lenses was 0.39 D (95% CI 0.21, 0.56; *p* < 0.001) and for spectacle lenses was 0.62 D (95% CI 0.27, 0.97; *p* < 0.001; Figure 2). The lowest heterogeneity was found in the atropine treatment subgroup (*I^2^ =* 54%) and the highest in the spectacle lenses subgroup (*I*^2^ *=* 100%). The axial length elongation MD for atropine was −0.12 mm (95% CI −0.15, −0.08; *p* = 0.04), for soft contact lenses was −0.18 mm (95% CI −0.26, 0.11; *p* < 0.001), for spectacle lenses was −0.34 mm (95% CI −0.35, −0.33; *p* < 0.001) and ortho-k lenses was −0.24 mm (95% CI −0.33 to −0.15; Figure 3). The lowest heterogeneity was found in the spectacle lenses treatment subgroup (*I*^2^ *=* 41%) and the highest in the soft contact lenses subgroup (*I*^2^ *=* 88%).

The following interventions were found to be effective in the reduction of myopia progression with statistical significance (*p* < 0.001): highly aspherical lenslets (HAL, refraction MD: 0.80, 95% CI 0.77–0.83; axial length: −0.35 mm, 95% CI -0.36 to −0.34), MiSight contact lenses (refraction MD: 0.66 D, 95% CI 0.63–0.69; axial length MD: −0.28 mm, 95% CI -0.29 to −0.27), low dose atropine 0.05% (refraction MD: 0.54 D, 95% CI 0.38–0.70; axial length MD: −0.21 mm, 95% CI-0.28 to −0.14), Biofinity +2.50 D (refraction MD: 0.45, 95% CI 0.29, 0.61; axial length: −0.24 mm, 95% CI -0.33 to −0.15), DIMS (refraction MD: 0.44, 95% CI 0.42–0.46; axial length: −0.34, 95% CI −0.35 to −0.33) and ortho-k lenses (axial length: −0.24 mm, 95% CI −0.33 to −0.15; Figures 2, 3).

Other interventions were also found to be effective but with lower effect sizes, such as extended depth of focus contact lenses, low dose atropine 0.025% or the esencia contact lens. The overall treatment effect was 0.37 D (95% 0.27–0.47) and −0.18 mm (95% −0.22 to −0.14). Low-dose atropine of 0.01% seemed to be the least effective method in controlling progression of myopia and axial length. Low-dose atropine 0.01% was not effective in reducing AL progression in 2 of the included studies (13, 22). There was high heterogeneity among treatment comparisons (*I*^2^ > 90%).

For topical low dose atropine, control effects reported as percentage reduction in progression ranged from 27% (0.01%) to 67% (0.05%) for myopia progression and from 12% (0.01%) to 51% (0.05%) for axial length elongation. Spectacle lenses such as DIMS and aspherical lenslets were effective in slowing myopia progression (percentage reduction of 87 and 67%, respectively) and axial elongation (percentage reduction of 61 and 64%, respectively) in children compared with controls. MiSight contact lenses (59% reduction in myopia progression and 52% reduction of axial elongation) and the Bifocal Lenses Biofinity +2.50 D also showed a significant slowing of myopia progression (reduction of 43%) and axial length elongation (reduction of 36%).

**Figure 4:**
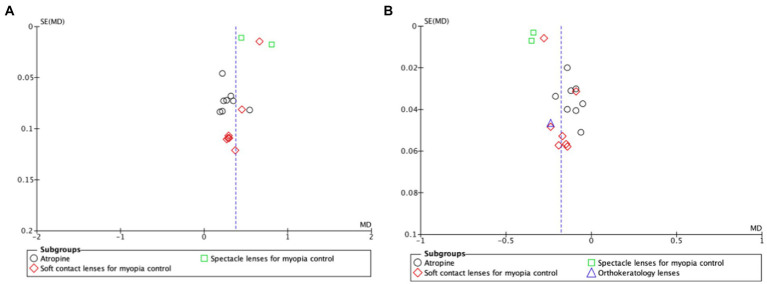
Funnel plot of comparison for myopia progression **(A)** and axial length elongation **(B)**. The funnel plot is a scatter plot that shows the effect estimates on the x-axis and measures of study precision (or study size) on the y-axis. The blue dotted vertical line represents the estimated common effect. SE, standard error; MD, mean difference.

Funnel plots for myopia progression and axial elongation shown in Figures 4A,B, respectively, found no publication biases.

## 4. Discussion

This study investigated the effectiveness of interventions to control myopia progression. In addition, previous reviews such as the IMI-white papers (31) were updated by providing a meta-analysis of treatment effect sizes. The following interventions were found to be effective in the reduction of myopia progression: HAL, MiSight contact lenses, low dose atropine 0.05%, Biofinity +2.50 D, DIMS and ortho-k lenses. Other interventions were also found to be effective but with lower effect sizes, such as extended depth of focus contact lenses and low dose atropine 0.025%. Low-dose atropine of 0.01% seemed to be the least effective method in the control of myopia progression and axial length elongation. Low-dose atropine 0.01% was not effective in the reduction of axial length elongation in two of the included studies.

Previous reviews concluded that there is high-level evidence to support the use of atropine to prevent myopia progression (7, 9, 11, 32). Those conclusions are consistent with our observations in this systematic review. The LAMP study (1-year) showed that topical atropine, even at low doses, remained one of the most effective treatments in slowing myopia progression in children aged 4–12 years (14). Although, concentrations of 1% are effective, there are associated side effects such as photophobia, as well as increases in myopia progression and axial length elongation following the cessation of treatment (rebound effect) (7). The results of our review showed that 0.01% seems to have less influence on axial elongation in Asian populations compared to 0.05% atropine that showed good efficacy and tolerability. Nevertheless, it is important to note that around 10% of children are non-responders and still have myopia progression even on high-dose atropine (33). In the 2-year follow-up of the LAMP study, 0.05% atropine remained the most effective concentration in the control of myopia progression (15). During the third year follow-up, children on continued treatment showed better myopia control results compared with children on the washout regimen (16). Nevertheless, the rebound phenomenon was small across the three atropine concentrations (0.05, 0.025, and 0.01%). Based on the 3-year trial results it seems that treatment should be ceased at an older age and that lower concentrations have smaller rebound effect. In fact, previous systematic-reviews and meta-analysis have found that low-dose atropine 0.01% showed good efficacy in controlling myopia progression (7, 17). Although low dosage atropine of 0.025 and 0.01% showed less effect in controlling myopia in some reported studies, the LAMP study also reported that myopia progression was effectively prevented by low dosage atropine 0.05, 0.025, and 0.01% among children with older age. Younger children required the highest 0.05% concentration to achieve similar reduction in myopic progression as older children receiving lower concentrations (34). Therefore, although more evidence and data are still needed, among the tested concentrations, 0.05% atropine may be optimal for children with older age.

However, the authors of those studies highlighted some limitations, such as the low sample size in some of the included studies, the reduced number of studies that evaluated the efficacy of 0.05% atropine and that most studies were conducted in Asia. Thus, findings may not be generalized to other ethnicities. Although, low-dose atropine is widely used in some East Asian countries for treating children with myopia, it has not been tested in European populations. Atropine is not commercially available in any of the European countries since clinical trials are still ongoing. There are 3 ongoing randomized trials in Europe, 2 in France and 1 in the United Kingdom, registered at the clinicaltrials.gov website. Differences between Asian and European populations are likely, given the well-known effects of iris pigmentation in relation to cycloplegic agents, such as atropine. A report on the efficacy of atropine arising from racial differences showed that atropine is less effective in populations of European than East Asian origin (35). New data also suggest that topical atropine treatment may be affected by environmental factors, such as extended time indoors. In a recent study from Israel children aged 9–15 years, under 0.01% atropine treatment (*n* = 14) had an increase in myopia progression and axial length during the COVID-19 lockdown year compared with the pre-lockdown year where the treatment was more effective (36).

The evidence regarding multifocal spectacles is evolving with time and the availability of new designs to slow myopia progression. Although a previous Cochrane systematic review concluded that there was no clinical meaningful slowing of eye growing (28), a more recent systematic review confirmed that multifocal lens have positive effects in slowing myopia progression both at 6 and 12 months with sustained effects until 36 months (8). Recent RCTs showed that multifocal lenses, either spectacles (HAL 2-year and DIMS 3-year including children aged 8–13 years) or contact lenses (MiSight 3-year and extended depth of focus 2-year including children aged 8–13 years), may confer a similar treatment benefit compared to atropine, with evidence of efficacy to slow both axial length and myopia progression in both Asian and European populations (9, 10, 14, 21, 26, 34, 35). HAL lenses (2-year) study were able to slow myopia progression by 0.80 D and axial length progression by 0.35 mm compared with children wearing single vision spectacle lenses (29). The myopia control efficacy was higher in children who wore their lenses full-time (≥12 h/day). Those results provide further proof of principle that devices such as HAL (based on the principle of imposing simultaneously a corrected image and a myopically blurred vision) slow the rate of myopia progression. This approach is further supported by the results obtained with the MiSight contact lenses (59% reduction in myopia progression and 52% reduction of axial elongation), which operate under the same principle (21). Children on the MiSight contact lenses trial were invited to continue the study for 3 additional years (MiSight 6-year). The results showed that MiSight contact lenses slowed the progression of myopia over a period of 6 years with a total reduction of 71% over the subsequent 3-year treatment period (22). The Bifocal Lenses Biofinity +2.50 D including children aged 7 to 11 years also showed a significant slowing of myopia progression (reduction of 43%) and axial length elongation (reduction of 36%) in a 3-year randomized trial (23). It should be noted that the MiSight lenses were approved by the US FDA for myopia control in children, and the Stellest lenses (HAL) were granted breakthrough status in 2021, which facilitates clinical use.

The results of our review showed that HAL-2 year, DIMS 3-year and MiSight 3-year seem to be more effective than orthokeratology contact lenses (18 months) in slowing axial elongation. In Scandinavian children aged 6–12 years orthokeratology lenses reduced AL elongation by 0.24 mm after 18-months follow-up without vision-threatening adverse events (30). However, most contact lenses and orthokeratology lenses (except for Menicon Bloom) are not approved for myopia control in Europe (off-label). Extended depth of focus soft lens are now available in some markets from Mark’ennovy (MYLO lens) and are CE marked for myopia management (37). Prescribing contact lenses in children is associated with risk of microbial keratitis. However, the risk is less (1 in 66 likelihood) than the risk of developing visual impairment due to complications of high myopia (1 in 5 likelihood), making contact lenses a worthy option for myopia control (38).

A study suggested that as a general goal, myopia control interventions should aim to provide a cumulative treatment effect of 1 D reduction to keep myopia below 6 D and axial length below 26 mm (40% less lifetime risk of developing myopic maculopathy) (39). Nevertheless, if a child progresses from −0.50 D in the early years of primary school, she or he will be highly myopic of −8.0 to −9.0 D by the end of schooling and 1 D reduction will not avoid the development of high myopia. With the new optical methods giving above 50% reduction in both spherical equivalent and axial length change over at least 3 years, eye care providers can aim for higher myopia reductions by incorporating myopic control into the first correcting spectacles to a child.

There are studies using combination of therapies for myopia control. Using topical 0.01% atropine with orthokeratology lenses has led to decreases in axial length elongation with most improvement during the first 6 months–1 year of treatment (40, 41). In another study, combining atropine 0.01% with orthokeratology was effective in children with baseline myopia of 1 to 3D, but no treatment benefit was found for children with higher baseline myopia (42). Nevertheless, the efficacy of this combined therapy was confirmed by two meta-analysis (43, 44). The interpretation of the results of those meta-analyses should take into consideration that the number of included studies was small and some studies were classified as having a high risk of bias. Thus, further research with well-designed RCT studies is important to understand if the treatment effect can be sustained over a longer follow-up period.

Although most of the treatment protocols seem to control progression of myopia, a few factors should be considered when analyzing the results. Most studies that tested treatment efficacy, recruited a small number of children and some children were lost-to-follow-up. For example, in the 3-year RCT of MiSight Lenses only 75.5% of the children concluded the study (53 MiSight children and 56 controls), in the ortho-k study 30% of the subjects dropped out before the treatment was well established and in the extended depth of focus study 25% of children discontinued the treatment soon after lens dispensing and prior to the 1-month visit (14, 35, 36). It is also important to note that refraction differences between the controls and experimental groups seem to diminish with time. Another limitation is the number of years of follow-up. One interesting point for discussion is the subgroup analysis and the covariate distribution such as the number of trials and participants contributing to each subgroup. Plausibility of interaction or lack of interaction, and possibility of confounding are important issues. Thus, further research is necessary.

Research on myopia control has increased over time with the number of publications increasing 4 times more from 1999 (almost 500 publications) to 2022 (about 2000 publications; www.pubmed.ncbi.nlm.nih.gov). Nevertheless, there is still no valid scientific criteria to decide when to initiate treatment based on progression and further research is necessary. Most pediatric ophthalmologists will treat children based on the rate of myopia progression (45). However, the ability to predict future myopia progression solely based on the rate of progression was found to be modest (46). The decision to treat should also be based on other factors, such as age of onset, ethnicity, parental myopia, axial length, and refraction at a given age. Different myopia progression risk calculators have been developed. Some will soon be available with new diagnostic devices designed to address the needs of myopia monitoring, usually based on autorefraction combined with biometry, and sometimes corneal topography (47).

When evaluating myopic progression and axial length elongation in treated children, it is important to analyze, for example, dose in the case of topical atropine, or when to discontinue treatment. For example, in children with myopia progression on low-dose atropine, the dose could be increased (0.01% twice a day; or 0.05, 0.1, 0.5%, or 1%). The decision must take into consideration that eye growth varies by season throughout the year, and it may be influenced by environmental factors (48, 49). Consequently there is the need to take at least a full year observation to keep track of environmental variables, such as outdoor time and near work (26, 33). Based on the 3-year trial results of the LAMP study (16) it seems that treatment can be continued until teenage years and later discontinued while monitoring the child for at least 12 months to avoid a rebound effect.

Our study has several limitations that should be highlighted. We only included 12 studies from 2019 to 2021. Although most studies were conducted in Asia, the target population varied. Both placebo, single vision spectacle lenses and single vision contact lenses were used as controls. These factors may have potential influence in our results. Thus, risk of bias cannot be excluded. There was high heterogeneity among each treatment regimen (*I*^2^ > 50%). Age was similar between studies and there was not sufficient data to explore how treatment varies with age.

The present study provides recent estimates of the efficacy of several therapies available to treat myopia progression by using data from 12 studies published over the last 3 years. This study also provides data on treatment comparisons that allows eye care providers to access the results and decide the best treatment options based on their efficacy and availability.

Further studies should focus on the effects of prolonged therapy taking in consideration the rebound phenomenon that is still present for some of the therapies. It is also important to determine the role of ethnicity in myopia treatment efficacy. Myopic macular degeneration has emerged as one of the leading causes of blindness and it is unclear how childhood myopia progresses into pathologic myopia in adulthood. Based on many ongoing experimental studies to control myopia progression, we can expect many new therapies to appear in the near future, and possibly some would stop myopia progression by 100%. The effective prevention of myopia onset is also awaited.

## 5. Conclusion

There is increasing evidence of myopia control by treatment protocols with proven efficacy. Based on evidence from the available RCTs reported in this analysis, the following evidence-based guidelines may be proposed: (1) HAL, MiSight contact lenses, low dose atropine 0.05%, Biofinity +2.50 D lenses, DIMS and ortho-k lenses were effective in the control of myopia progression; (2) Low dose atropine 0.025% and extended depth of focus contact lenses have also been found to be effective, but with lower effect sizes; (3) Low-dose atropine 0.01% was not as effective in reducing axial length progression according to some Asian studies. The recent data on new optical treatments, including soft contact lenses, DIMS and HAL, leads to optimism as these methods have shown considerable efficacy. Since they are much less invasive than alternatives such as orthokeratology and atropine, they are in principle likely to be preferable options. However, these results need to be confirmed in future as current knowledge is limited in the length of study periods and number of populations studied.

## Author contributions

AG was responsible for the concept and design of the study. CL performed the data acquisition and analysis, as well as interpreting the results and drafting the manuscript. CP participated in interpretation of results and revision of the manuscript. All authors contributed to the article and approved the submitted version.

## Conflict of interest

The authors declare that the research was conducted in the absence of any commercial or financial relationships that could be construed as a potential conflict of interest.

## Publisher’s note

All claims expressed in this article are solely those of the authors and do not necessarily represent those of their affiliated organizations, or those of the publisher, the editors and the reviewers. Any product that may be evaluated in this article, or claim that may be made by its manufacturer, is not guaranteed or endorsed by the publisher.
