# Corrigendum: Effectiveness of myopia control interventions: a systematic review of 12 randomized control trials published between 2019 and 2021

**DOI:** 10.3389/fpubh.2024.1460156

**Published:** 2024-09-25

**Authors:** Carla Lanca, Chi Pui Pang, Andrzej Grzybowski

**Affiliations:** ^1^Escola Superior de Tecnologia da Saúde de Lisboa (ESTeSL), Instituto Politécnico de Lisboa, Lisboa, Portugal; ^2^Comprehensive Health Research Center (CHRC), Escola Nacional de Saúde Pública, Universidade Nova de Lisboa, Lisboa, Portugal; ^3^Department of Ophthalmology and Visual Sciences, The Chinese University of Hong Kong, Hong Kong, China; ^4^Hong Kong Hub of Paediatric Excellence, The Chinese University of Hong Kong, Hong Kong, China; ^5^Joint Shantou International Eye Center, Shantou University/The Chinese University of Hong Kong, Shantou, China; ^6^Department of Ophthalmology, University of Warmia and Mazury, Olsztyn, Poland; ^7^Institute for Research in Ophthalmology, Foundation for Ophthalmology Development, Poznan, Poland

**Keywords:** myopia, progression, axial length, elongation, treatment, efficacy, systematic review

In the published article, there was an error in Figure 2 as published. The legend inside Figure 2 was incorrect. “Favours [control]” and “Favours [experimental]” were swapped. The corrected Figure 2 and its caption appear below.

**Figure 2:**
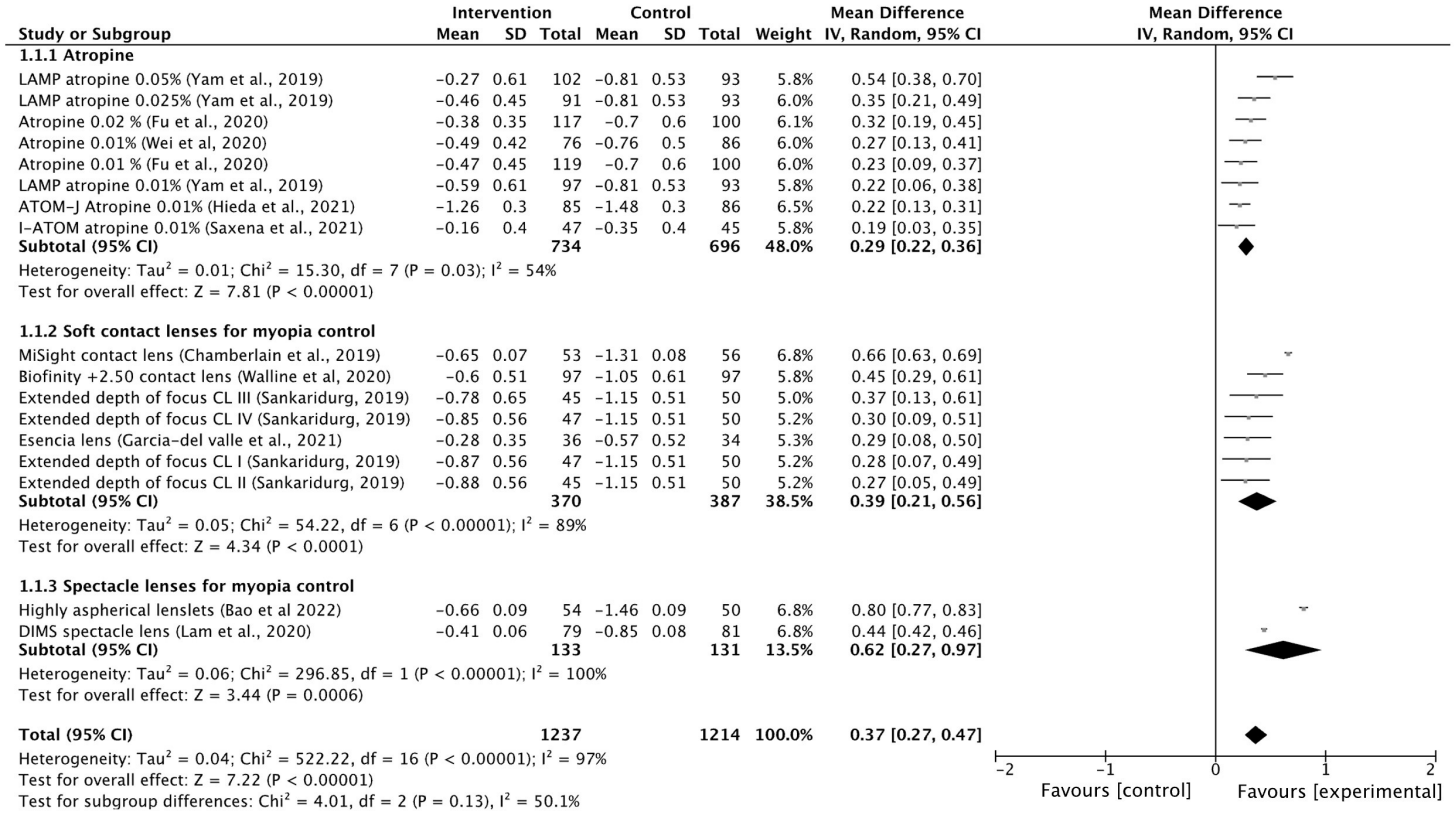
Forest plot of myopia progression (D) showing mean differences between treatment and control groups. The point estimate for the mean difference for each study is shown in gray color. The weight assigned to each study is represented by the size of each gray point estimate. The horizontal line through each gray point estimate shows the 95% confidence interval for the mean difference for each treatment. CL, contact lenses; CI, confidence interval; SD, standard deviation.

The authors apologize for this error and state that this does not change the scientific conclusions of the article in any way. The original article has been updated.

